# Supplementary material for: Waste to Medicine: Evidence from Computational Studies on the Modulatory Role of Corn Silk on the Therapeutic Targets Implicated in Type 2 Diabetes Mellitus
Source: Biology (Basel). 2023 Dec 11;12(12):1509. doi: 10.3390/biology12121509 (PMC10740667; doi:10.3390/biology12121509)
Supplement: Supplementary file 1 [file biology-12-01509-s001.zip › biology-2729436-supplementary.pdf]

Supplementary materials

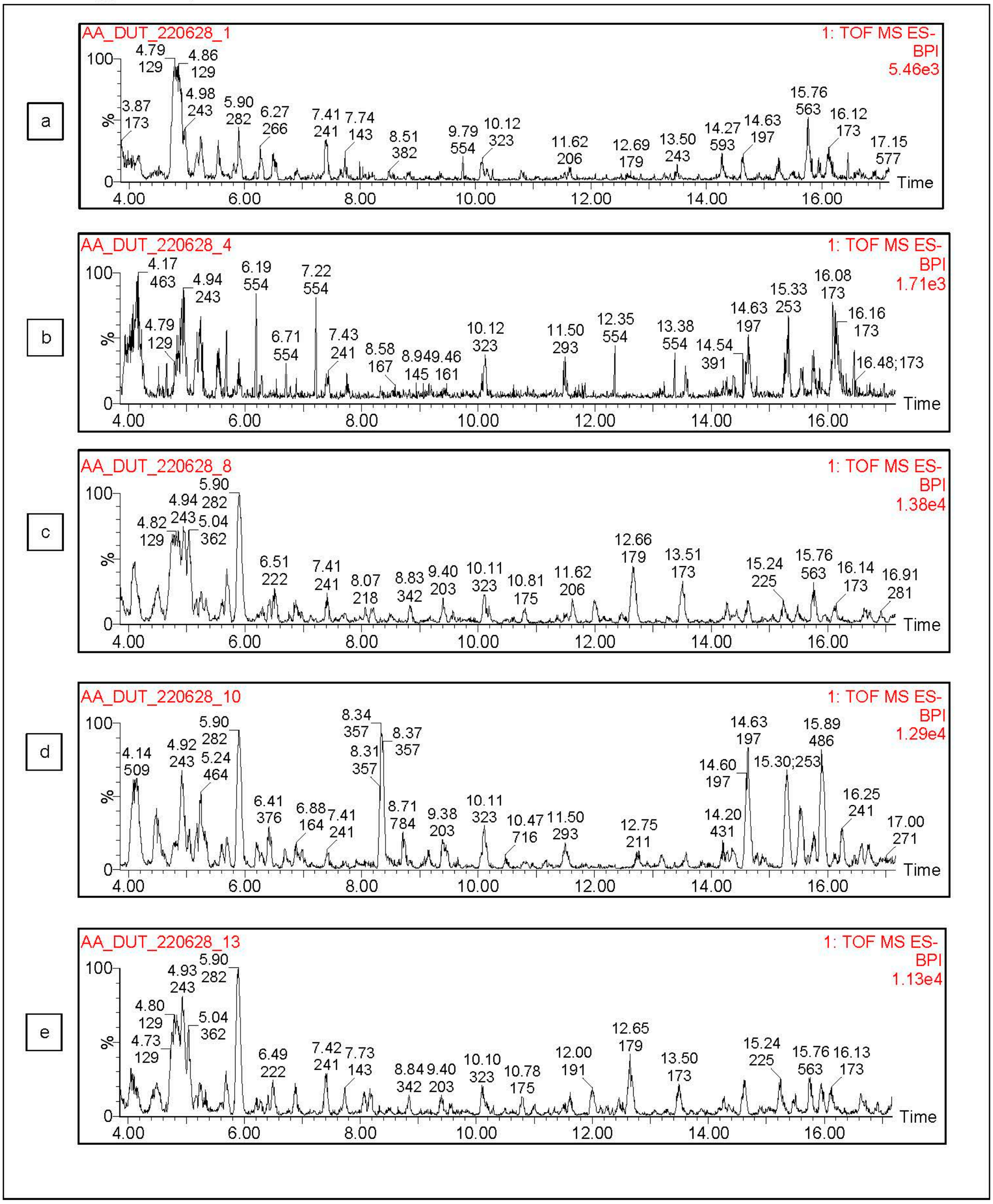

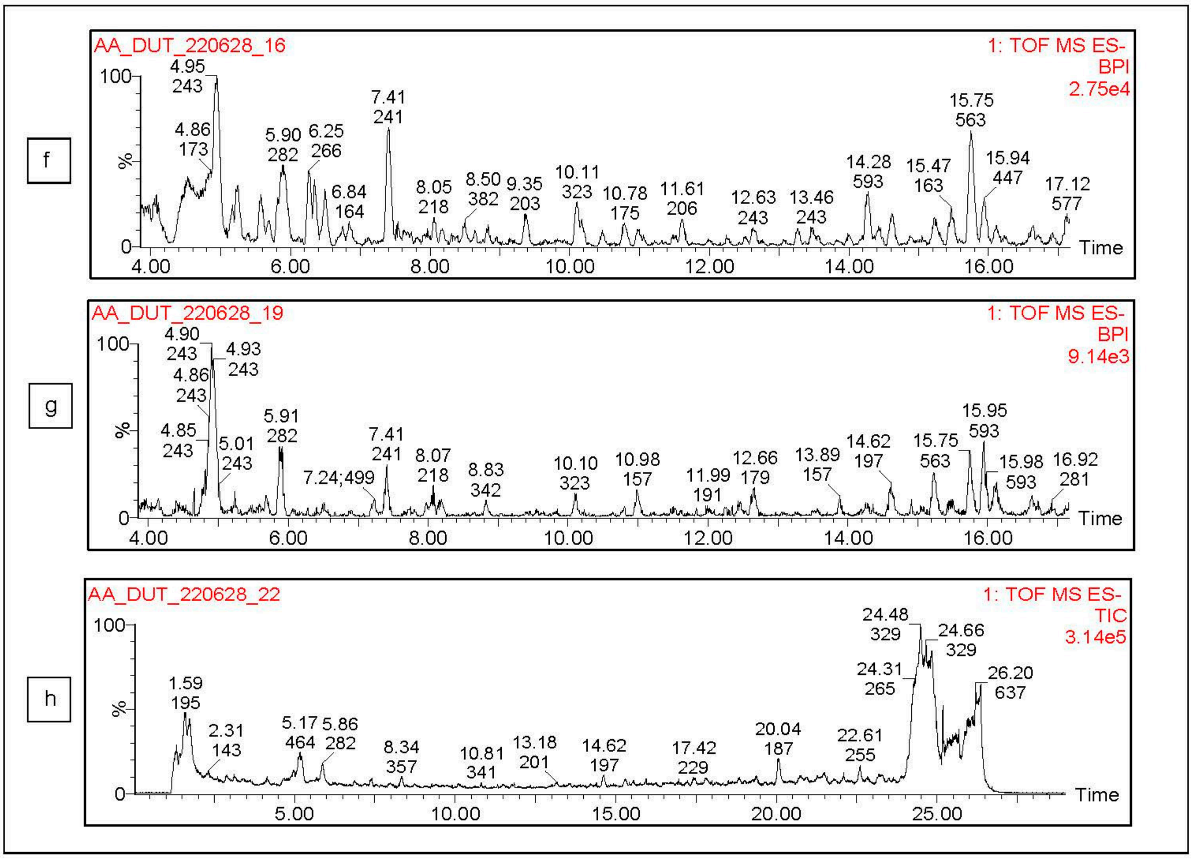

Figure S1: Examples of chromatograms produced showing mass to charge and retention times of compounds found in a) raw premature CS, b) raw mature cs as well as CS extracts; c) aqueous premature, d) aqueous mature, e) hydroethanolic premature, f) hydroethanolic mature, g) ethanolic premature and h) ethanolic mature.

Table S1: Bioactive secondary metabolites identified in various extracts of CS through UPLC-MS analysis

Compound number	Identity	Average retention time (minute)	m/z [M - H]-
1	(7'R)-(+)-Lyoniresinol 9'-glucoside	4,071	581,2203
2	Estriol-16-Glucuronide	4,149	509,2002
3	Ascorbic acid	4,307	175,0235
4	Methylisocitric acid	4,389	205,0338
5	Citraconic acid	4,524	129,0178
6	Oxalosuccinic acid	4,984	189,0053
7	Pinocembrin 7-apiosyl-(1->5)-apiosyl-(1->2)-glucoside;5,7-Dihydroxyflavanone 7-apiosyl-(1->5)-apiosyl-(1->2)-glucoside	4,988	681,2069
8	Mevalonic acid	5,089	147,0657
9	D-2-Hydroxyglutaric acid	5,176	147,0287
10	(2R,3S)-2,3-dimethylmalic acid	5,236	161,0445
11	UNPD109129	6,348	251,0772
12	Glutaric acid	6,152	131,0349
13	Cinnamic acid	6,849	147,0448
14	(6E)-1-(4-hydroxyphenyl)-7-phenylhepta-4,6-dien-3-one	8,452	277,1251
15	Vanillic acid	8,553	167,0338
16	1-O-vanilloyl-beta-D-glucose	8,553	329,0892
17	Isorhamnetin 3-(6''-malonylglucoside)	8,642	563,1026
18	Saccharumoside C	9,081	461,1286
19	7-hydroxy-4-{3-oxo-3H-benzo[f]chromen-2-yl}-2H-chromen-2-one	9,14	355,0631
20	Sarmentose	9,447	161,0809
21	CNP0447999	9,571	353,084
22	1-O-Caffeoylglucose	9,75	341,0882
23	1-O-Galloylglycerol	10,05	243,0488
24	C00051555	10,106	323,1328
25	3-Isopropylmalate	10,789	175,0606
26	Diaportinic acid	10,819	279,0534
27	Benzyl beta-D-glucopyranoside;(-)-Benzyl-O-beta-D-glucopyranoside	11,147	269,103
28	Domesticoside	11,354	343,1013
29	Dihydrophaseic acid 4'-O-beta-D-glucopyranoside	11,369	443,1937
30	Ketoleucine	11,471	129,0549
31	Aesculin	11,471	339,0728
32	Chlorogenic acid	11,997	353,0874
33	Quinic acid	12,004	191,055
34	Cudraphenone A	12,229	363,1627
35	Pimelic acid	12,255	159,0653
36	(-)-6-((2S,3R,4R,5S,6R)-3,4-dihydroxy-6-(hydroxymethyl)-5-methoxytetrahydro-2H-pyran-2-yloxy)-8-hydroxy-3-methyl-1H-isochromen-1-one	12,458	367,1035

37	(-)-11-hydroxy-9,10-dihydrojasmonic acid 11-beta-D-glucoside	12,515	389,1799
38	Caffeic acid	12,653	179,0349
39	5-Carboxyvanillic acid	12,729	211,0234
40	Phellodendric acid A;(+)-Phellodendric acid A	12,754	203,0944
41	(-)-Epigallocatechin	13,267	305,0676
42	D-Leucic acid	13,289	131,0713
43	cis-Aconitic acid	13,501	173,0094
44	MINEs-320976	14,199	431,1909
45	Loganic acid	14,248	375,1276
46	Astragalin 7-rhamnoside	14,269	593,1522
47	Quing hau sau; quinghaosu; artemisinin	14,353	281,1399
48	3-Hydroxy-4-butanolide	14,432	263,0786
49	Annularin A;(+)-Annularin A	14,623	197,081
50	Traumatic acid	14,781	227,1267
51	Methyl geranate	14,869	181,1236
52	3-Hydroxytetradecanedioic acid	14,873	273,1726
53	Dihydrophaseic acid	14,937	281,1376
54	Tuberonic acid	15,238	225,1111
55	Daldiniapyrone	15,294	253,1071
56	4-Hydroxycinnamic acid	15,479	163,0386
57	Monascusone A	15,536	253,106
58	Acetovanillone;Apocynin	15,596	165,0547
59	Apiin	15,756	563,1376
60	Quercitrin	15,953	447,0915
61	2-Hydroxydecanedioic acid	16,176	217,1065
62	UNPD221406	16,241	241,1083
63	Cichorioside M;(+)-Cichorioside M	16,618	427,1946
64	Austricin;8-Deacetylmatricarin;Desacetylmatricarin	16,714	261,1154
65	trans-Ferulic acid	17,086	193,0499
66	UNPD19396	17,122	577,15
67	UNPD77208	17,208	271,1529
68	Nepetaside	17,236	345,1552
69	Dodecanedioic acid	17,417	229,1427
70	Kaempferitrin	17,554	577,1528
71	Chrysoeriol 4',7-diglucuronide	17,675	651,118
72	erythronolide B	17,734	401,2548
73	UNPD230015	17,818	263,0574
74	3-Hydroxysebacic acid	17,836	217,106
75	Tricin 7-diglucuronoside	17,958	681,1317
76	Esculetin	18,079	177,0172
77	ethyl 5-[(2,5-dimethylphenyl)methoxy]-2-phenyl-1-benzofuran-3-carboxylate	18,087	399,1613
78	xi-2,2,6-Trimethyl-1,4-cyclohexanedione	18,254	153,0928
79	Quercetin 3-(2'',3'',4''-triacetylgalactoside)	18,307	589,1204
80	[4]-Gingerol	18,844	265,1428
81	Phaseic acid	18,558	279,1234
82	Diaportinol	18,603	265,0729
83	Herbacetin 7-(6''-quinoylglucoside)	18,677	637,1406

84	12-deoxyerythronolide A	19,046	401,253
85	UNPD156113	19,094	605,1201
86	Mirificin	19,148	547,1448
87	Phaseolic acid	19,244	261,1326
88	Curvularol	19,367	265,1433
89	(+)-(S)-Carvone	19,645	149,0958
90	Kaempferol 3-rhamnoside 7-galacturonide	19,868	607,1331
91	Azelaic acid	20,074	187,0968
92	Kaempferol 3-rhamnoside 7-galacturonide	20,286	607,1347
93	Stagonolide F;(-)-Stagonolide F	20,677	183,1045
94	Isowertin 2''-rhamnoside	20,732	1183,344
95	UNPD223815	20,864	255,1598
96	9-hydroxynonanoic acid	20,885	173,1173
97	Maysin	20,941	1151,286
98	5,7,4'-Trihydroxy-3'-methoxyflavone; Luteolin 3'-methyl ether 7-glucuronide	21,005	475,085
99	Quercetin 3-O-(6''-acetyl-glucoside)	21,144	505,0988
100	Blennin D	21,154	265,1425
101	10-Deoxygeniposidic acid	21,271	357,1225
102	Gallicynoic acid F;(-)-Gallicynoic acid F	21,409	343,2093
103	Pandangolide 1a;(-)-Pandangolide 1a	21,459	243,1215
104	4-Hydroxynonenal	21,569	155,1078
105	Tetradecanedioic acid	21,764	257,1747
106	(R)-7-butyl-6,8-dihydroxy-3-[(3E)-pent-3-en-1-yl]-3,4-dihydroisochromen-1-one	21,818	303,1627
107	Genistin	21,815	431,0959
108	p-Coumaroyl malic acid	20,852	282,1126
109	Kaempferol 3-[2'''-acetyl-alpha-L-arabinopyranosyl-(1->6)-galactoside]	21,942	621,1427
110	Cyperine	22,091	259,0962
111	(+)-Cnicin; Cnicin	22,111	377,1621
112	(S)-Abscisic acid	22,202	263,1284
113	Sydnic acid; Sydonic acid	22,233	265,1424
114	Gallicynoic acid A;(+)-Gallicynoic acid A	22,806	253,1445
115	Apigenin 7-O-(6''-O-acetylglucoside)	22,847	473,1057
116	UNPD127537	23,18	255,1597
117	Olivetol	23,205	179,1065
118	Ginsenoyne E	23,293	303,1604
119	Caffeoyl tartaric acid	23,326	312,1225
120	Luteolin 3'-methyl ether 7-glucuronide	23,476	265,1425
121	UNPD205010	23,478	343,1748
122	Maysin 3'-methyl ether	23,505	589,1541
123	Artemin	23,657	265,1458
124	Gallicynoic acid B	23,687	267,159
125	Aspergillide A	23,804	253,1443
126	(S)-Oleuropeic acid	23,889	183,1018
127	Sebacic acid	23,894	201,1113
128	7-Acetoxy-5,6-dimethoxycoumarin	17,818	263,0574

a

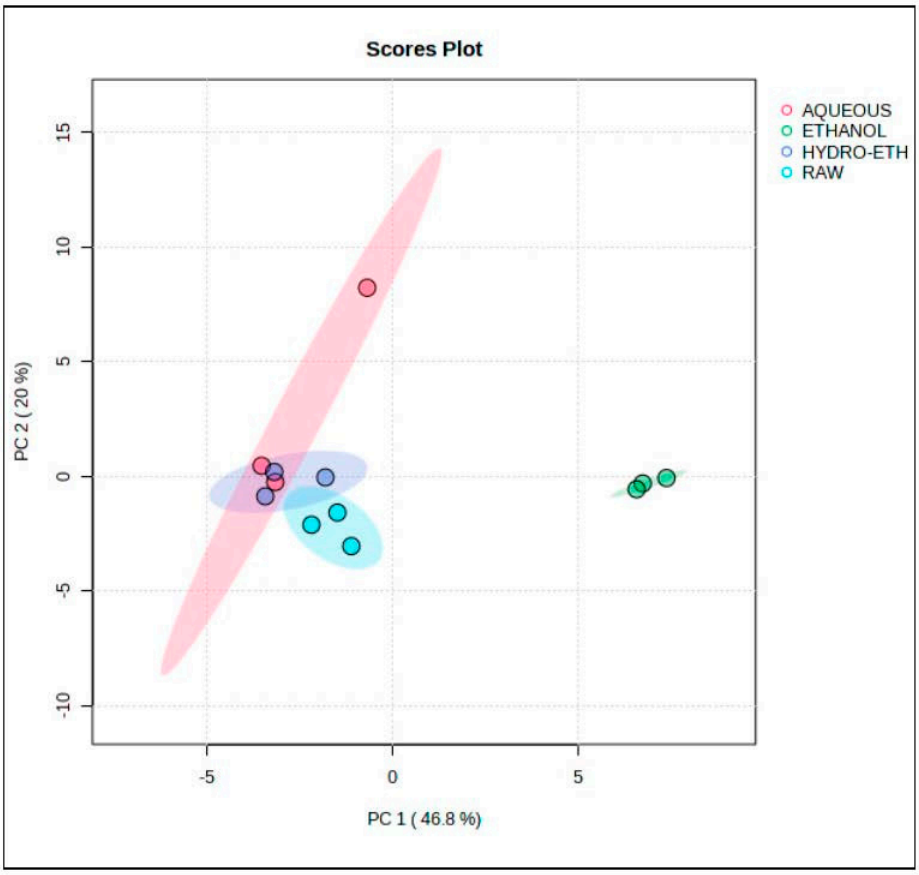

Scores Plot
AQUEOUS
ETHANOL
HYDRO-ETH
RAW
PC 2 (20 %)
PC 1 (46.8 %)
15
10
5
0
-5
-10
-5
0
5

b

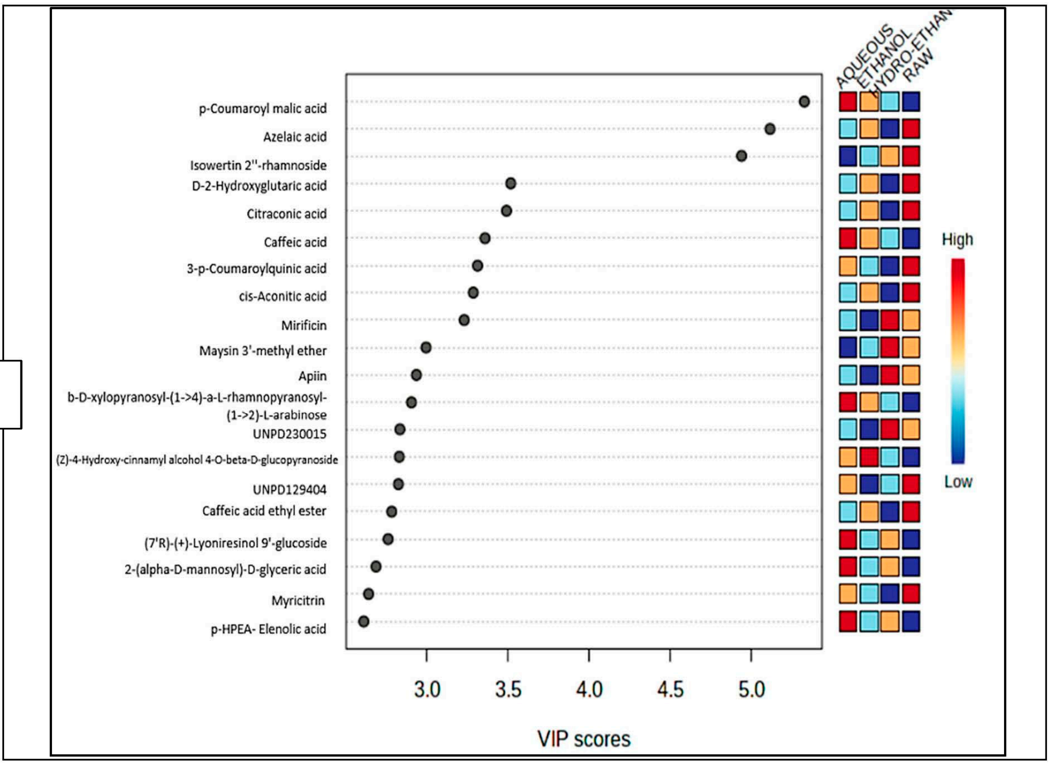

AQUEOUS
ETHANOL
HYDRO-ETHAN
RAW
p-Coumaroyl malic acid
Azelaic acid
Isowertin 2''-rhamnoside
D-2-Hydroxyglutaric acid
Citraconic acid
Caffeic acid
3-p-Coumaroylquinic acid
cis-Aconitic acid
Mirificin
Maysin 3'-methyl ether
Apiin
b-D-xylopyranosyl-(1->4)-a-L-rhamnopyranosyl-(1->2)-L-arabinose
UNPD230015
(Z)-4-Hydroxy-cinnamyl alcohol 4-O-beta-D-glucopyranoside
UNPD129404
Caffeic acid ethyl ester
(7'R)-(+)-Lyoniresinol 9'-glucoside
2-(alpha-D-mannosyl)-D-glyceric acid
Myricitrin
p-HPEA- Elenolic acid
High
Low
3.0
3.5
4.0
4.5
5.0
VIP scores

Figure S2: a) Principal component analysis scores plot showing variance in CS secondary metabolites between the four extracts; b) Partial least square discriminant analysis (PLS-DA) loadings plot showing the differences in the amount of secondary metabolites present in the four samples of CS.

Table S2: Number of Lipinski's violations of CS bioactive compounds

	Formula	Molecular weight (g/mol)	#H-bond acceptors	#H-bond donors	iLOGP (octanol/ water partition coefficient)	Lipinski #violations
1	C28H38O13	582.59	13	7	4.71	3
2	C24H32O9	464.51	9	6	1.67	1
3	C6H8O6	176.12	6	4	0.39	0
4	C7H10O7	206.15	7	4	-0.94	0
5	C5H6O4	130.10	4	2	0.46	0
6	C6H6O7	190.11	7	3	-0.71	0
7	C31H38O17	682.62	17	9	2.27	3
8	C6H12O4	148.16	4	3	0.39	0
9	C5H8O5	148.11	5	3	0.18	0
10	C6H10O5	162.14	5	3	0.34	0
11	C9H16O8	252.22	8	5	0.37	0
12	C5H8O4	132.11	4	2	0.45	0
13	C9H8O2	148.16	2	1	1.55	0
14	C19H18O2	278.35	2	1	2.79	0
15	C8H8O4	168.15	4	2	1.40	0
16	C14H18O9	330.29	9	5	1.88	0
17	C25H24O15	564.45	15	7	1.77	3
18	C19H26O13	462.40	13	7	1.35	2
19	C22H12O5	356.33	5	1	2.52	0
20	C7H14O4	162.18	4	2	0.94	0
21	C23H14O4	354.35	4	1	2.41	0
22	C15H18O9	342.30	9	6	0.23	1
23	C10H12O7	244.20	7	5	0.63	0
24	C13H24O9	324.32	9	5	1.95	0
25	C7H12O5	176.17	5	3	0.24	0
26	C13H12O7	280.23	7	3	1.54	0
27	C13H18O6	270.28	6	4	1.60	0
28	C15H20O9	344.31	9	5	2.05	0
29	C21H32O10	444.47	10	6	1.89	1
30	C6H10O3	130.14	3	1	1.06	0
31	C15H16O9	340.28	9	5	1.04	0
32	C16H18O9	354.31	9	6	0.96	1
33	C7H12O6	192.17	6	5	-0.12	0
34	C23H24O4	364.43	4	2	3.70	0
35	C7H12O4	160.17	4	2	0.94	0
36	C17H20O9	368.34	9	4	2.54	0
37	C18H30O9	390.43	9	5	2.35	0
38	C9H8O4	180.16	4	3	0.97	0
39	C9H8O6	212.16	6	3	0.24	0
40	C9H16O5	204.22	5	2	1.55	0
41	C15H14O7	306.27	7	6	0.98	1
42	C6H12O3	132.16	3	2	1.17	0

43	C6H6O6	174.11	6	3	-0.13	0
44	C20H32O10	432.46	10	6	1.57	1
45	C16H24O10	376.36	10	6	1.26	1
46	C27H30O15	594.52	15	9	1.80	3
47	C15H22O5	282.33	5	0	2.75	0
48	C10H16O8	264.23	8	4	0.97	0
49	C10H14O4	198.22	4	1	2.32	0
50	C12H20O4	228.28	4	2	2.04	0
51	C11H18O2	182.26	2	0	3.05	0
52	C14H26O5	274.35	5	3	2.34	0
53	C15H22O5	282.33	5	3	2.09	0
54	C12H18O4	226.27	4	2	1.46	0
55	C13H18O5	254.28	5	0	3.07	0
56	C9H8O3	164.16	3	2	0.95	0
57	C13H18O5	254.28	5	3	1.82	0
58	C9H10O3	166.17	3	1	1.77	0
59	C26H28O14	564.49	14	8	2.11	3
60	C21H20O11	448.38	11	7	1.27	2
61	C10H18O5	218.25	5	3	0.96	0
62	C12H18O5	242.27	5	2	1.81	0
63	C21H32O9	428.47	9	5	1.97	0
64	C15H18O4	262.30	4	1	2.03	0
65	C10H10O4	194.18	4	2	1.62	0
66	C34H26O9	578.56	9	6	3.45	2
67	C14H24O5	272.34	5	1	2.71	0
68	C16H26O8	346.37	8	4	2.06	0
69	C12H22O4	230.30	4	2	1.83	0
70	C27H30O14	578.52	14	8	1.89	3
71	C28H28O18	652.51	18	9	0.94	3
72	C21H38O7	402.52	7	4	2.83	0
73	C13H12O6	264.23	6	3	1.70	0
74	C10H18O5	218.25	5	3	1.32	0
75	C29H30O19	682.54	19	9	1.95	3
76	C9H6O4	178.14	4	2	1.25	0
77	C26H24O4	400.47	4	0	4.45	1
78	C9H14O2	154.21	2	0	1.63	0
79	C27H26O15	590.49	15	5	1.94	2
80	C15H22O4	266.33	4	2	2.38	0
81	C15H20O5	280.32	5	2	1.76	0
82	C13H14O6	266.25	6	3	2.33	0
83	C28H30O17	638.53	17	11	1.81	3
84	C21H38O7	402.52	7	4	2.83	0
85	C27H26O16	606.49	16	9	2.59	3
86	C26H28O13	548.49	13	8	0.66	3
87	C12H22O6	262.30	6	4	1.32	0
88	C15H22O4	266.33	4	2	2.35	0
89	C10H14O	150.22	1	0	2.27	0
90	C27H28O16	608.50	16	9	1.66	3
91	C9H16O4	188.22	4	2	1.44	0
92	C27H28O16	608.50	16	9	1.66	3
93	C10H16O3	184.23	3	1	2.03	0
94	C28H32O14	592.55	14	8	0.80	3
95	C14H24O4	256.34	4	1	3.26	0
96	C9H18O3	174.24	3	2	1.83	0

97	C27H28O14	576.50	14	8	1.95	3
98	C22H20O12	476.39	12	6	2.02	2
99	C23H22O13	506.41	13	7	1.61	3
100	C15H22O4	266.33	4	2	2.46	0
101	C16H22O9	358.34	9	5	1.24	0
102	C18H32O6	344.44	6	5	2.95	0
103	C12H20O5	244.28	5	2	1.91	0
104	C9H16O2	156.22	2	1	2.15	0
105	C14H26O4	258.35	4	2	2.49	0
106	C18H24O4	304.38	4	2	3.45	0
107	C21H20O10	432.38	10	6	2.11	1
108	C13H12O7	280.23	7	3	0.62	0
109	C28H30O16	622.53	16	8	1.81	3
110	C15H16O4	260.29	4	2	2.73	0
111	C20H26O7	378.42	7	3	1.97	0
112	C15H20O4	264.32	4	2	1.97	0
113	C15H22O4	266.33	4	3	2.59	0
114	C14H22O4	254.32	4	3	2.19	0
115	C23H22O11	474.41	11	5	2.31	1
116	C14H24O4	256.34	4	2	2.66	0
117	C11H16O2	180.24	2	2	2.04	0
118	C17H22O2	258.36	2	0	4.10	0
119	C13H12O9	312.23	9	5	0.55	0
120	C28H30O16	622.50	4	3	2.59	1
121	C17H28O7	344.40	7	2	2.59	0
122	C28H30O14	590.53	14	7	1.76	3
123	C15H22O4	266.33	4	2	2.19	0
124	C15H24O4	268.35	4	3	2.87	0
125	C14H22O4	254.32	4	1	2.59	0
126	C10H16O3	184.23	3	2	1.65	0
127	C10H18O4	202.25	4	2	1.77	0
128	C13H12O6	264.23	6	3	1.70	1

Table S3: Frontier molecular orbitals for the top-scoring compounds against ADORA1, HCAR2, GABBR1

Compound	Frontier molecular orbital
ADORA1	

Cyperine	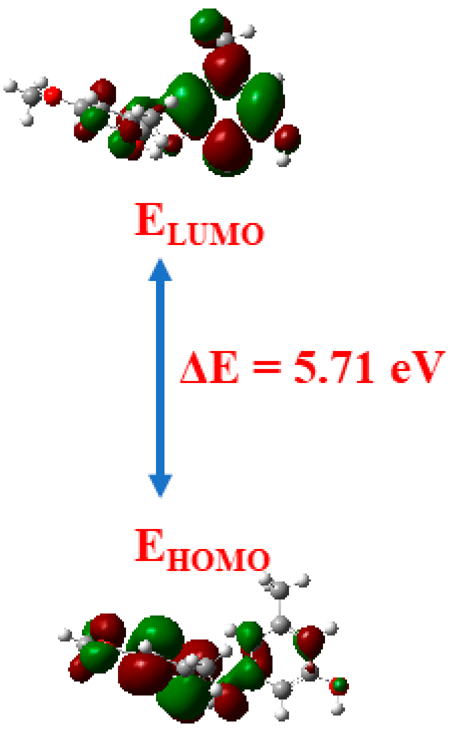
Domesticoside	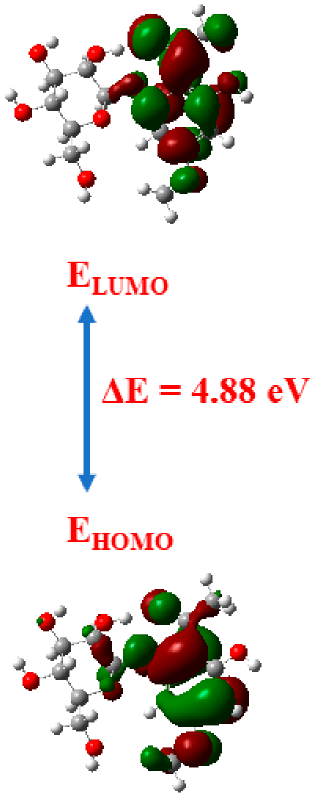

Gallicynoic acid B	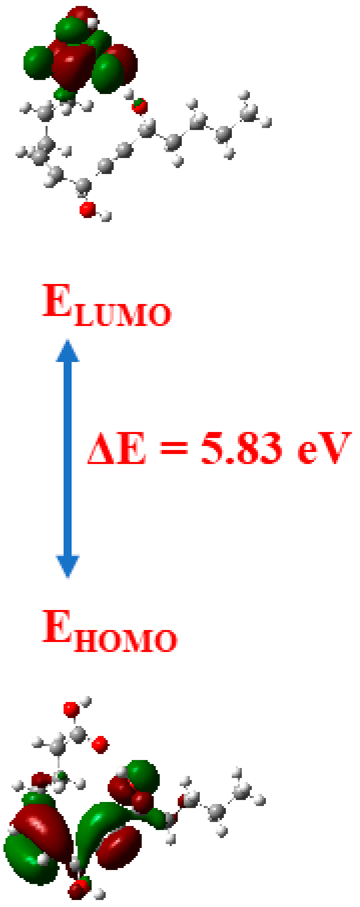

Ginsenoyne E	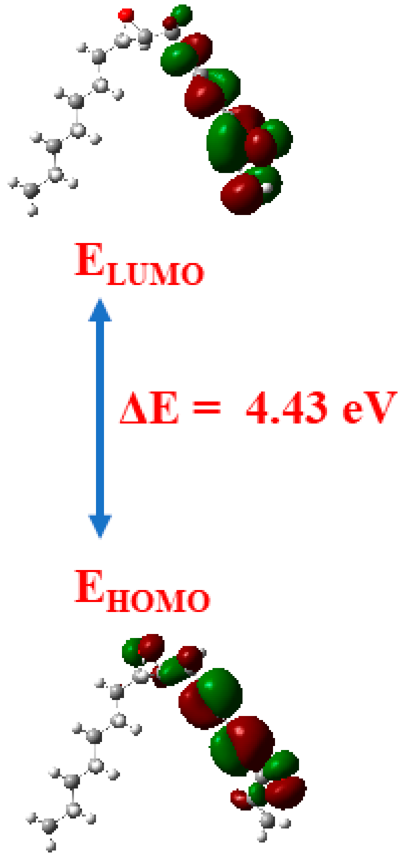
Quing hau sau	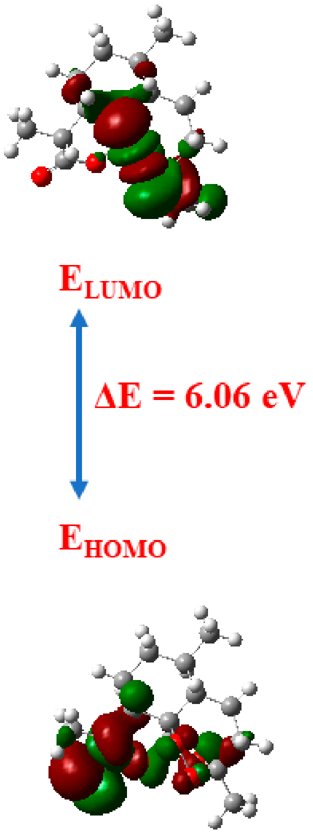

Metformin	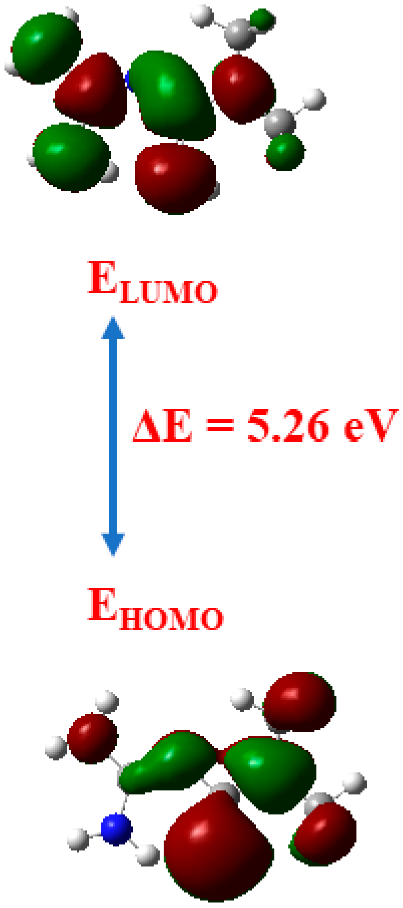
Reservatrol	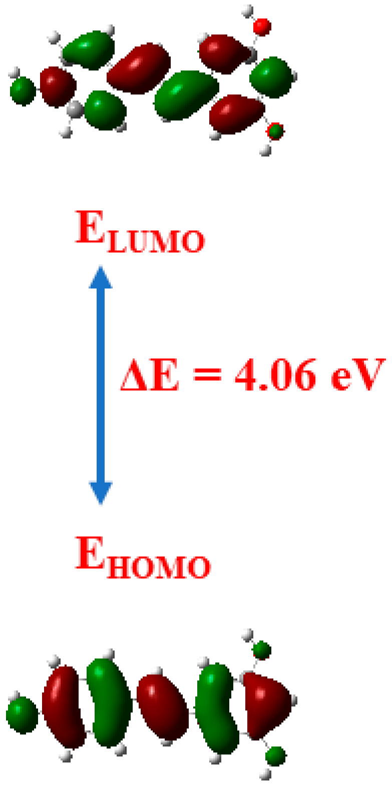

**HCAR2**	
Caffeic acid	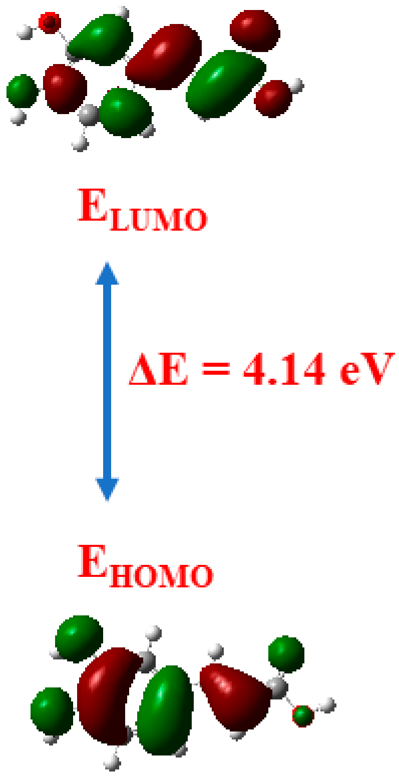
Dodecanedioc acid	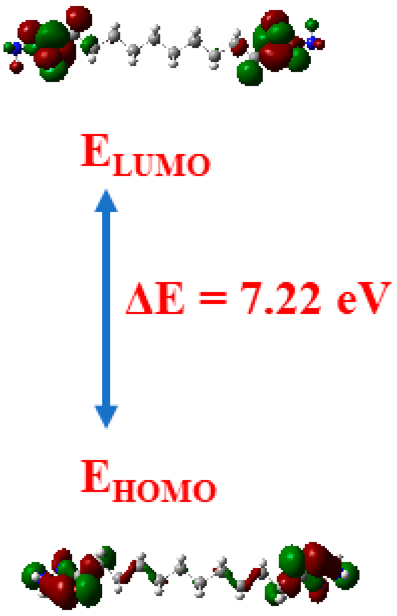

4-Hydroxycinnamic acid

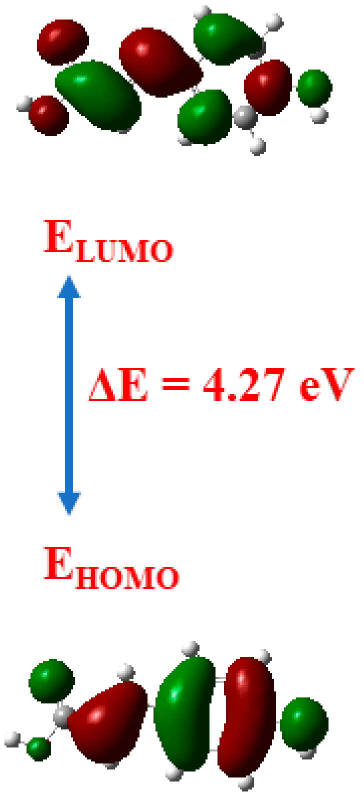

Phaseic acid

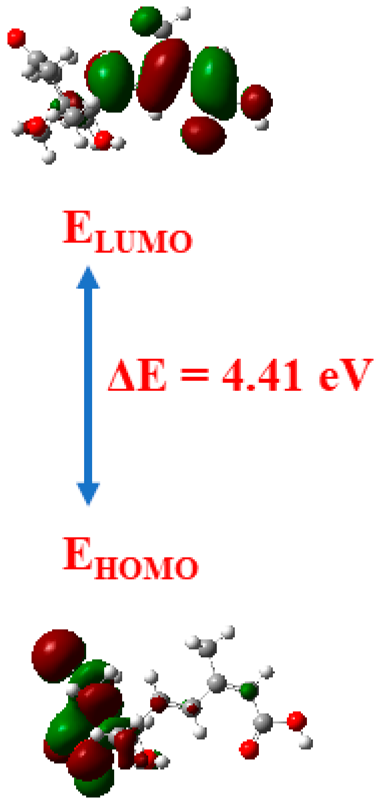

Sebaic acid	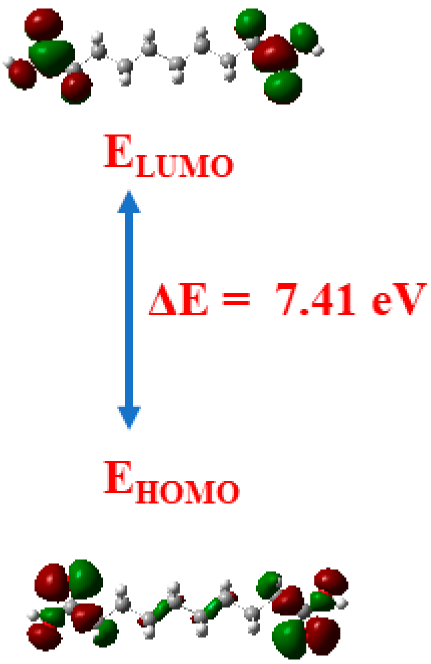
Metformin	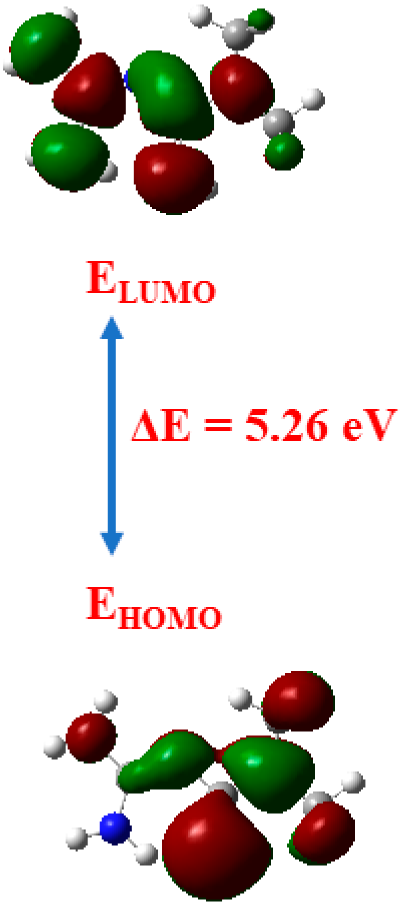

<table>
<tr><td>Reservatrol</td><td>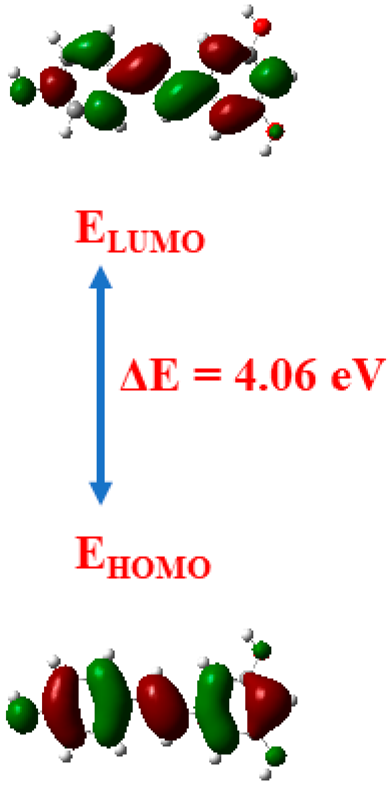
</td></tr>
<tr><td colspan="2">GABBR1</td></tr>
<tr><td>Dodecanedioc acid</td><td>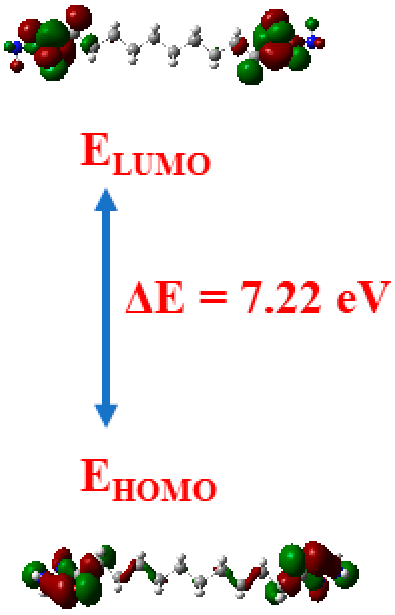
</td></tr>
</table>

Methylisocitric acid	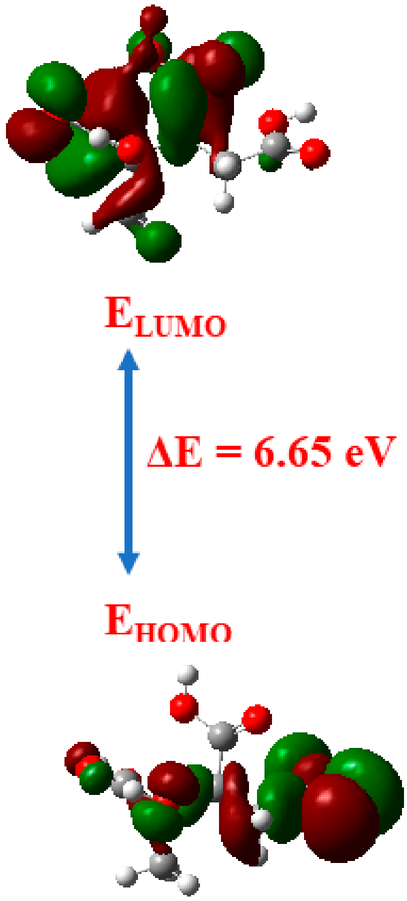

Quinic acid	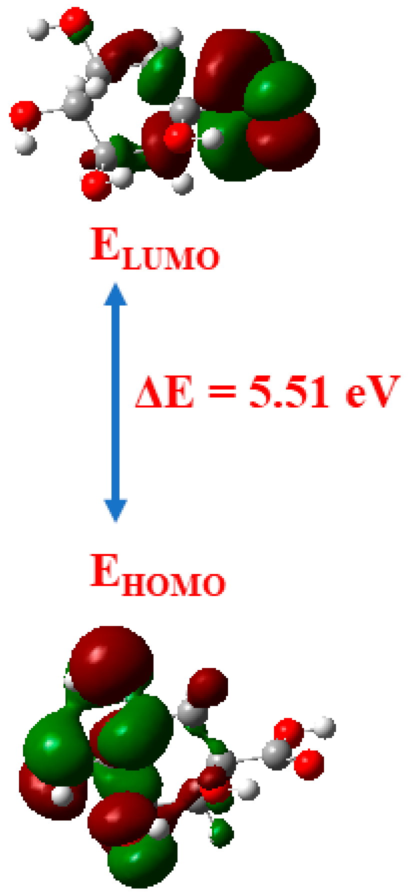
Tetradecanedioc acid	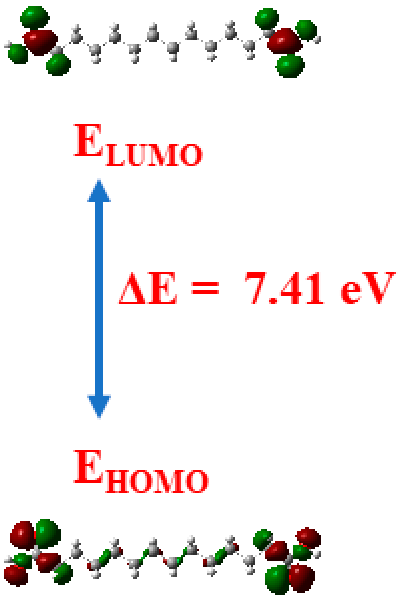

Xi-2,2,6-Trimethyl-1,4-Cyclohexanedione	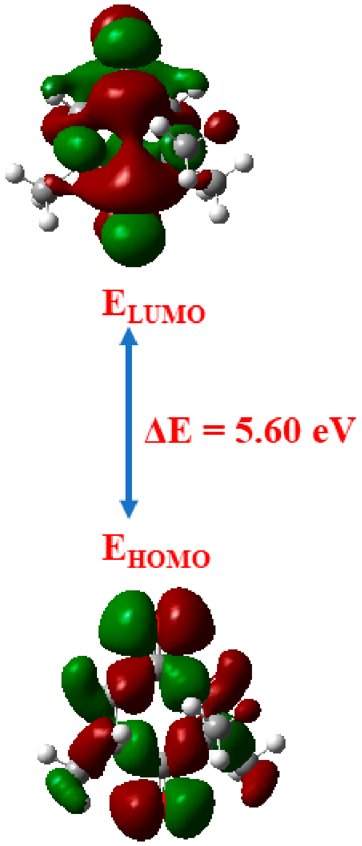

Metformin	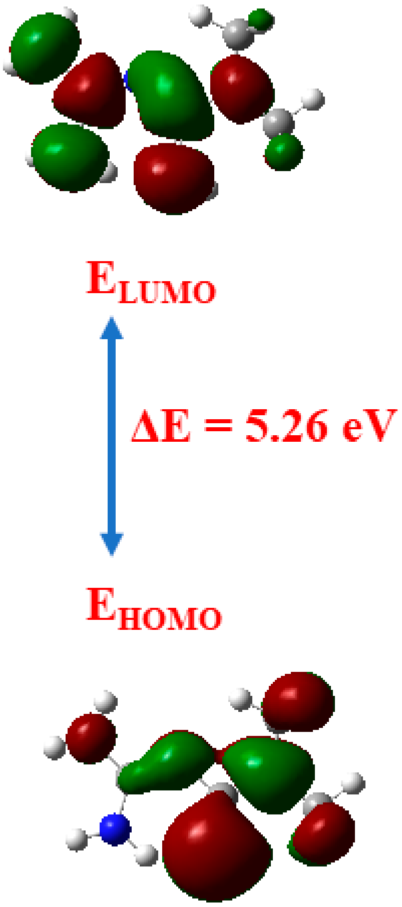
Reservatrol	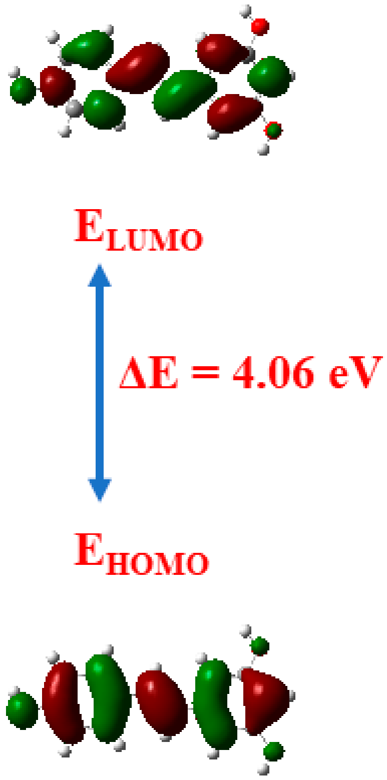

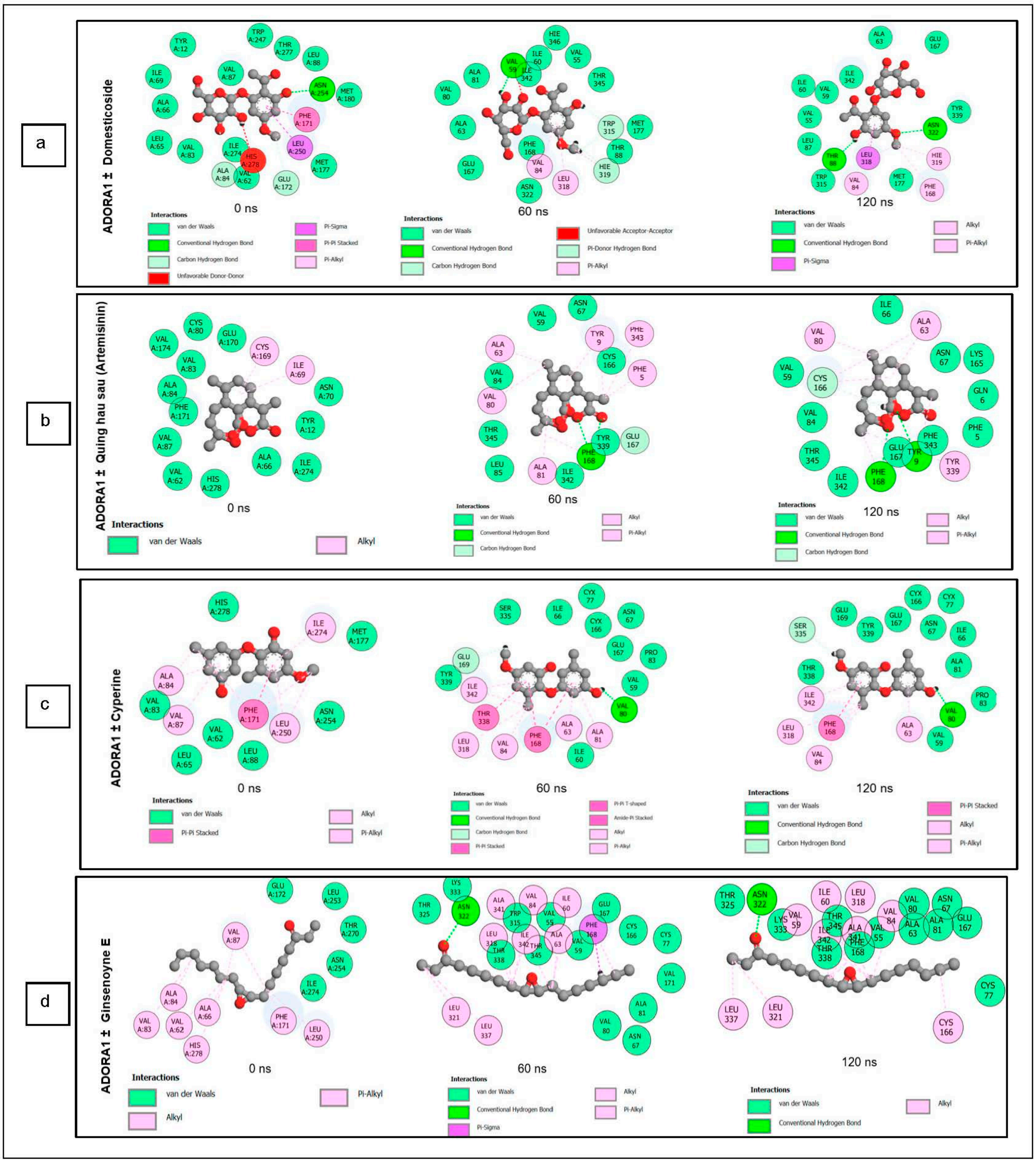

Figure S3: a) domesticoside, b) quing hau sau, c) cyperine and d) ginsenoyne E, over a 120 ns simulation

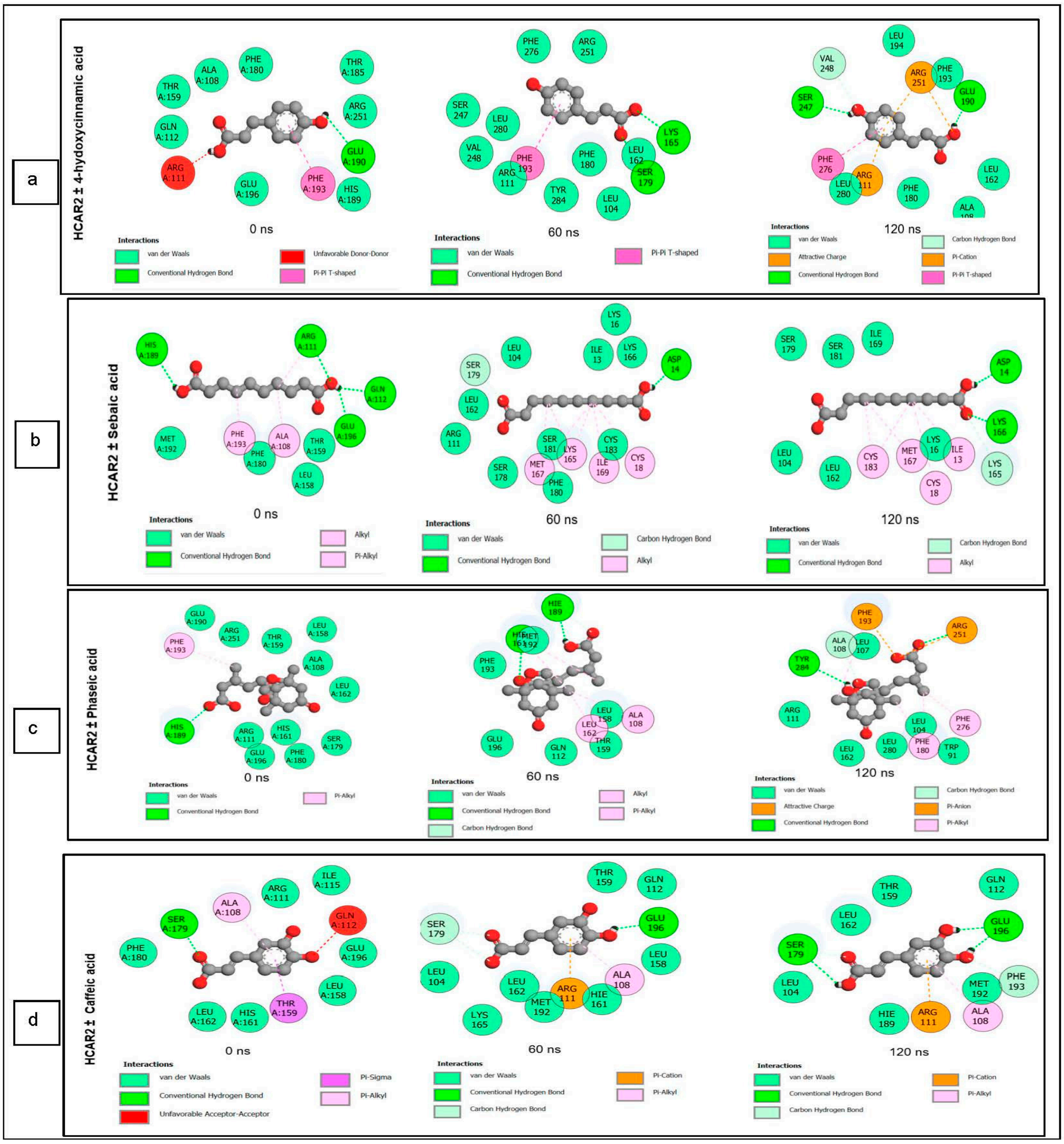

Figure S4: 2-D interaction plots of target gene HCAR2 with bioactive constituents present in CS: a) 4-hydroxycinnamic acid, b) sebacic acid, c) phaseic acid and d) caffeic acid over a 120 ns simulation

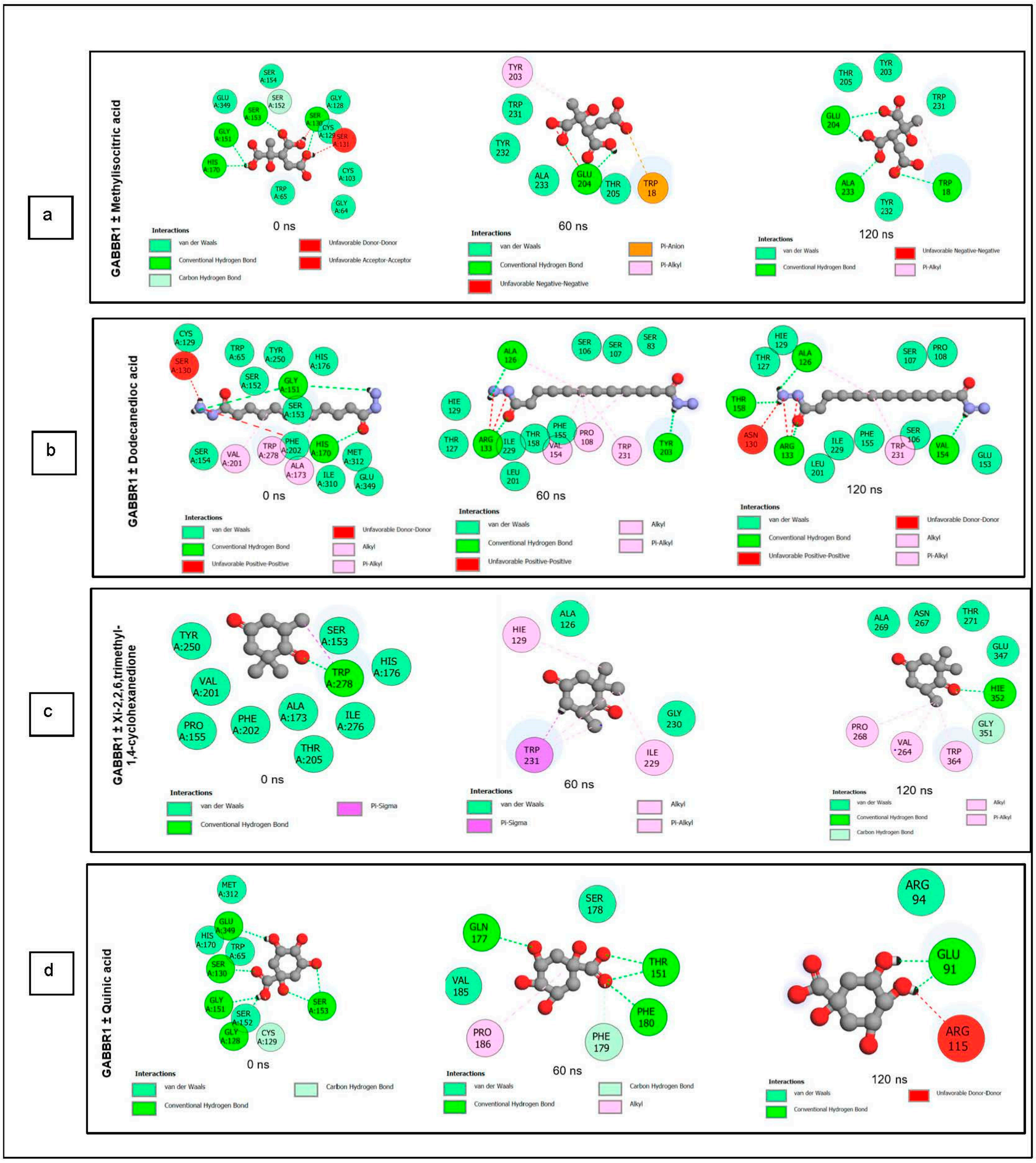

Figure S5: 2-D interaction plots of target gene GABBR1 with bioactive constituents present in CS: a) methylisocitric acid, b) dodecanedioc acid, c) xi-2,2,6,trimethyl-1,4-cyclohexanedione and d) quinic acid, over a 120 ns simulation.
